# Radiologic comparison of posterior release, internal distraction, final PSO and spinal fusion with one-stage posterior vertebral column resection for multi-level severe congenital scoliosis

**DOI:** 10.1186/s12891-017-1627-9

**Published:** 2017-06-20

**Authors:** Shichang Liu, Nannan Zhang, Yueming Song, Zongrang Song, Liping Zhang, Jijun Liu, En Xie, Qining Wu, Dingjun Hao

**Affiliations:** 1 Department of Spine Surgery, Honghui Hospital, Xi’an Jiaotong University College of Medicine, South door slightly Friendship Road 555, Xi’an, 710000 People’s Republic of China; 2 National Center for Birth Defect Monitoring, West China Second University Hospital, Sichuan University and Key Laboratory of Birth Defects and Related Diseases of Women and Children (Sichuan University), Ministry of Education, Chengdu, Sichuan, 610041 People’s Republic of China; 3 Department of Orthopedics, West China Hospital, Sichuan University, Chengdu, People’s Republic of China

**Keywords:** Multi-level congenital scoliosis, Vertebral column resection, Posteriorrelease, Internal distraction

## Abstract

**Background:**

To compare radiologic results of posterior release, internal distraction, and final pedicle subtraction osteotomy (PSO) and spinal fusionwith one-stage posterior vertebral column resection (PVCR) in treating multi-level severe congenital scoliosis.

**Methods:**

Forty-onesevere congenital scoliosis patients were used in the study. Group A comprised 24 patients who underwent one-stage PVCR. Group B comprised 17 patients who underwent posterior release with internal distraction, followed by final posterior fusion and instrumentation. The average preoperative main curve was 110.4° (95–130°) in group A and 109.4° (range 90°–126°) in group B. Postoperative follow-up time was ≥2 years (2.0–4.5 years) to analyze the radiographic and clinical outcomes.

**Results:**

A comparison of posterior release, internal distraction, and final spinal fusion with PVCR showed no significant differences in postoperative main curve and compensatory caudal curve correction, coronal and sagittal imbalance. However, significant differences were found between the 2 groups in compensatory cranial curve correction.

**Conclusions:**

Posterior release, internal distraction, and final spinal fusion produce better corrective results in compensatory cranial curve correction than PVCR in treating severe multi-level congenital scoliosis.

## Background

The treatment of severe congenital spinal deformities is challenging. Conventional procedures, such as angular osteotomies, including Ponte, Smith–Peterson, and pedicle subtraction, might not provide the desired amount of correction in very severe deformities with coronal or sagittal plane decompensation. Over the last 10 years, posterior vertebral column resection (PVCR) has been described and popularized as an effective method by which to treat severe rigid congenital scoliosis [1–4]. As reported, a correction rate of 51–59% can be achieved by PVCR, but there is also a high neurological risk [5–9].

The growing rod technique is a relatively new method by which to facilitate curve correction through a safe, staged stretching of the spine. Some surgeons have used anterior or posterior release and growing rod technique to treat unbalanced congenital scoliosis with positive corrective results [10, 11]. Different from other types of severe scoliosis, the structural dysplasia and anterior release of multi-level severe congenital scoliosis may lead to spinal instability. Providing that the patients often suffer from poor lung function and the intraoperative turning over for posterior surgery may cause nerve damage, the posterior release procedure may be a better choice in treating severe congenital scoliosis.

Patients with severe multi-level congenital scoliosis are generally poor in physical condition and low tolerance for surgery, therefore, it is important to determine the most safe and effective method to treat this population. The purpose ofour study was to compare the radiological and clinicaloutcomes of one-stage PVCR and posterior release, internal distraction followed by posterior fusionandinstrumentationfor severe mutil-level congenital scoliosis. We examined the records of 41 patients with severe rigid congenital scoliosis who were treated in our hospital between 2009 and 2014, and compared the radiological and clinical outcomes of posterior release, internal distraction, and final spinal fusion with one-stage PVCR for severe multi-level congenital scoliosis.

## Methods

### Patients and evaluation

This was a retrospective analysis of 41 patients with severe multi-level congenital scoliosis (major curve Cobb angle ≥90°on films) treated in our hospital between 2009 and 2014. Group A comprised 24 patients who underwent one-stage PVCR and instrumentation. Group B comprised 17 patients who underwent posterior release with posterior internal distraction (with or without further distraction), followed by final PSO and fusion. The choosing of surgical methods in this study is based on the different surgeons’ own habits in our center. The average preoperative main curve for group A was 110.4° (95°–130°) and for group B was 109.4° (90°–126°). The patients were reviewed after a minimum follow-up of 2 years post-surgery (2.0–3.6 years). Clinical records were reviewed for complications, demographic data, radiological data, hospital expenses, and length of stay. Before surgery, standing anteroposterior lateral radiographs as well as supine right and left bending radiographs were taken to measure spinal column curve and flexibility. Standard preoperative imaging also included magnetic resonance imaging(MRI) and computed tomography(CT) scans of the spinal column and intrathecal structures. Cervical MRI was used to examine that there is no patient with Chiari malformations, nor pure syringomyelia, spinal cord tumors, split cord malformation. If the above-mentioned patients are found, they are transferred to the neurosurgical treatment, such as spinal cord drainage, posterior fossa reconstruction, intramedullary septal resection, and then operate the correction surgery of scoliosis to prevent nerve injury during this process. The patient evaluations were performed before surgery, after surgery, and during the most recent follow up. Radiographs were measured in coronal and sagittal planes for scoliosis, kyphosis, lordosis. Cervical MRI was used to exclude Chiari malformations, syringomyelia, spinal cord tumors and split cord malformation. If any of these disorders was detected, patients were transferred to the care of the neurosurgical department for appropriate treatment before scoliosis correction surgery, to reduce the risk of spinal cord injury. As the operating time between posterior release and final-stage fusion was relatively short, the blood loss was counted in the GR group as that lost in the one-stage posterior release and final-stage fusion combined. Blood loss in the PVCR group was defined as that lost during the whole procedure.

The segmental curve was measured as the angle of the upper and the lower end plates of the hemivertebra on the standing postero-anterior radiograph. The segmental curve was measured as the angle of the upper and the lower hemivertebral end plates on the standing postero-anterior radiograph. The main curve was measured as the Cobb angle of the two most tilted vertebrae. The compensatory cranial and caudal curves were also measured as the Cobb angles. Coronal balance was defined as the distance from the C7 plumb line to the central sacral line on the standing postero-anterior radiograph, while sagittal balance was defined as the distance from the C7 plumb line to the posterior superior corner of the body of S1 on the standing lateral radiograph. Lumbar lordosis was represented by the angle between the upper end plates of L1 and S1. Thoracic kyphosis was represented by the angle between the upper end plate of T5 and the lower end plate of T12.Two experienced surgeons independently measured each radiographandmean values were used statistic alanalysis.

### Surgical techniques

#### Posterior vertebral column resection

The surgical techniques was performed in accordance with the procedure described by Suk et al. [7]. The patient was placed in a prone position and a standard midline incision was made. The posterior elements of the spine were carefully exposed. Pedicle screws were inserted segmentally, except for in the resected levels. The posterior elements of the hemivertebra were removed, including the lamina, facet joints, transverse process, and posterior part of the pedicle. In the thoracic spine, the rib head and the proximal part of the surplus rib on the convex side was resected. Then, the lateral cortex of the hemivertebra was exposed by blunt dissection. Before removing the vertebral body, a stabilizing rod was then placed on one side (usually the concave side at coronal plane deformities). Careful subperiosteal dissection was performed on the working site (opposite the stabilizing rod) to follow the lateral wall of the vertebral body until the anterior aspect was easily palpable.

The pedicle, lateral aspect of the vertebral body and the discs above and below the planned resection level were then completely removed. After resection of the posterior wall on the working side, another temporary rod was inserted into the working side and securely locked to the screws with slight compression to shorten the vertebral column. The temporary rod on the opposite side was then removed to allow resection of the remaining vertebra. The same sequence of resection was performed on the remaining vertebra. A mesh cage was usually placed anteriorly to prevent too much shortening of the spinal column, which might cause duralbuckling, risking injury to the spinal cord. Gradual compression was applied while leaving the concave rod unlocked until the gap was closed. A final evaluation of the spinal canal was then conducted to confirm that there was no residual compression at the resected margins and no bony or disc tissue anterior to the dura. Decortication of the posterior elements was performed, and autogenous bone from the hemivertebra was used for fusion.

**Fig. 1:**
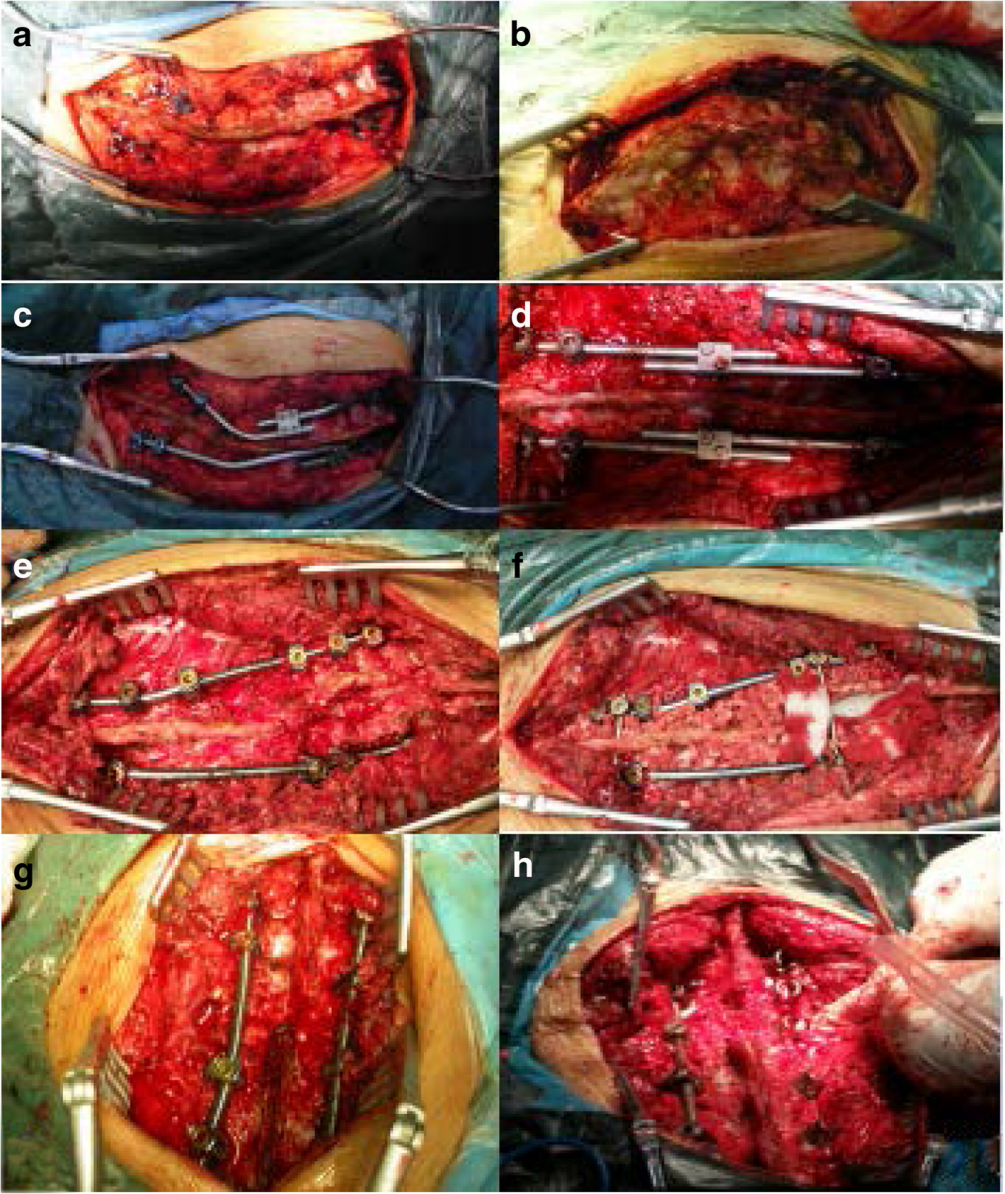
**a**-**b** Figures of intraoperative posterior release. **c**-**d** Figures after distraction of the growing rod. **e**-**f** Figures of the final surgery; pedicle subtraction osteotomy and spinal fusion. **g**-**h** Intraoperative figures; surgical procedures were based on the surgery proposed by Suk [24]

**Fig. 2:**
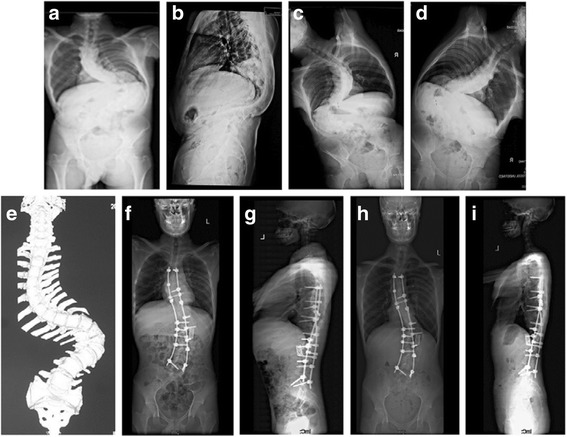
**a–e** An 11-year-old male with severe and rigid congenital kyphoscoliosis; **f**, **g**The patient underwent posterior vertebral column resection. Postoperatively, the Cobb angle was corrected from 130 to 60° with an acceptable coronal and sagittal balance; **h**, **i** At the 24-month follow up, no relevant loss of correction was observed

When the patient began to walk after surgery, a custom-made orthosis was used. The orthosis was kept for 6 months and removed if radiographs after 6 months showed no sign of pseudarthrosis (Figs. 1 and 2).

#### Posterior release and internal distraction

**Fig. 3:**
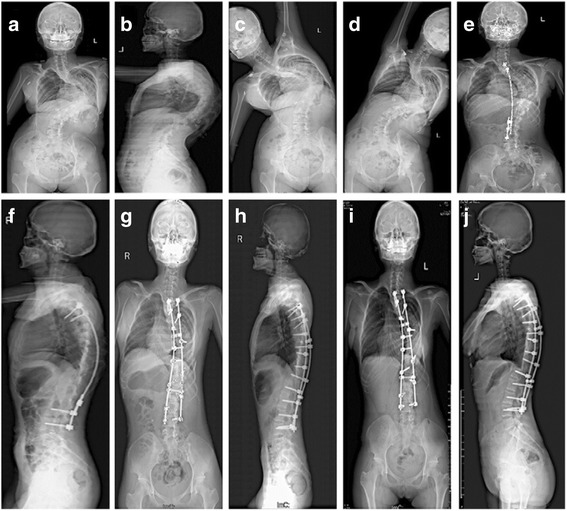
**a–d** A 13-year-old female with severe congenital scoliosis;**e**, **f** The patient underwent posterior release, internal distraction and final PSO and spinal fusion. The main curve was most corrected after the surgery of posterior release; **g**, **h**: After spinal fusion, the main curve were corrected to 22°; **i**, **j** At the 24-month follow up no relevant loss of correction in the main curve was observed

For posterior release and internal distraction surgery, the patient was placed in a prone position and a standard midline incision was made. Posterior exposure consisted of dissection of subcutaneous tissue and subperiosteal dissection of the spinous processes, laminae, and transverse processes. The release was extended throughout the ligament, beginning at the midline and proceeding laterally toward both facets. Dissecting toward the midline along the rib, the transverse process and rib head were exposed and resected along with approximately 3 cm of rib (costotransversectomy). The number of ribs resected depended on the characteristics of the scoliosis curve. This posterior release was performed along the main curve at each level. Then, two pedicle screws were inserted at the cephalad end and two at the caudal end of the major coronal curve on the concave side. A longer distraction rod connected to the cephalad fixation points was inserted subcutaneously, and a shorter distraction rod was connected to the caudal fixation points. These two rods were connected with a side-by-side (Domino) connector (Fig. 3) and were distracted by repeatedly loosening and tightening alternating screw nuts. If curve correction of at least 50% was not achieved with the first distraction procedure, an additional distraction procedure was performed 1–2 weeks after the initial surgery.

Approximately 1–4 weeks after the initial surgery or second distraction and when the patient’s medical status had stabilized, the final posterior spinal fusion was performed. This procedure was similar to that in the above PVCR fusion steps. The previously implanted pedicle screws were either retained or partially removed, according to the requirements for further correction.

### Analysis of data

Continuous variables were shown as mean ± standarddeviation. Statistical analysis was performed with SPSSversion 17.0 software. The intra-class correlation coefficient (ICC) was used to determine the inter-rater reliability. Clinical and radiological data before and after surgery were compared using pairedt-test. Two-sample *t* tests, Wilcoxon signed-rank test, or Pearson’s Chi-squared test were used to compare intergroup differences. A *p*-value < 0.05 was consideredsignificant.

## Results

Group A consisted of 24 patients (18 females and 6 males) and group B consisted of 17 patients (11 females and 6 males). A Student’s t-test found no significant differences in age (*P* = 0.175), post-surgery follow-up time (*P* = 0.717), and number of implant screws (*P* = 0.546). There are significant differences between two groups in the blood loss, mean length of stay and hospital expenses (Table 1).

**Table 1:** Demographics and Surgical Data on Patients

Variable	Group A (*n* = 24)	Group B (*n* = 17)	*P* value
Age (years)	15.6 ± 4.5(11-26)	14.3 ± 4.3(14–24)	0.175
Follow-up (months)	26.5 ± 5.3(24-38)	28.3 ± 5.8(24–42)	0.717
Blood loss (ml)	1754 ± 657.3	1168 ± 472.1	0.028
Screw number	14.3 ± 1.6(12-16)	14.6 ± 1.4(12–16)	0.546
Length of stay (days)	13.6 ± 3.2(11-21)	18.2 ± 4.2(14–26)	0.034

### Radiological results

The ICC showed excellent inter-rater values for both radiologic measurements (ranged from 0.78 to 0.91) (Table 2). Radiological results are presented in Table 3 and case examples are shown in Figs. 1 and 2. Two groups showed no significant differences in preoperative and postoperative coronal and sagittal imbalance, main curve and compensatory caudal curves correction.

**Table 2:** The intra-class correlation coefficient (ICC) analysis

Item	ICC(95% CI)
Segmental curve(°)	0.81(0.78-0.85)
Main curve(°)	0.78(0.70-0.83)
Compensatory cranial curve(°)	0.91(0.84-0.93)
Compensatory caudal curve (°)	0.81(0.77-0.84)
Coronal balance(cm)	0.83(0.79-0.88)
Sagittal balance(cm)	0.86(0.81-0.92)
T5-12 kyphosis(°)	0.78(0.71-0.82)
L1-S1 lordosis(°)	0.85(0.81-0.9)

**Table 3:** Radiological Results

Items	Group A (*n* = 24)	Group B (*n* = 17)	*P* value
Preoperative			
Segmental curve(°)	110.4 ± 10.5 (95-130)	109.4 ± 11.3 (90–126)	0.772
Main curve(°)	118.3 ± 9.8 (102-142)	114.1 ± 12.7 (100-133)	0.249
Compensatory cranial curve(°)	53.1 ± 6.5 (35-62)	50.7 ± 8.0 (36–61)	0.334
Compensatory caudal curve (°)	57.0 ± 6.6 (42-70)	58.1 ± 7.3 (41–69)	0.607
Coronal balance(cm)	0.8 ± 2.6 (−4.2 to 4.5)	0.2 ± 2.8 (−3.6 to 3.8)	0.853
Sagittal balance(cm)	0.2 ± 2.1 (−3.1 to 4.0)	0.4 ± 1.9 (−3.0 to 3.5)	0.711
T5-12 kyphosis(°)	64.2 ± 12.9 (33-85)	57.1 ± 15.9 (42-85)	0.202
L1-S1 lordosis(°)	−62.9 ± 10.2 (45-84)	−61.0 ± 15.3 (35-72)	0.638
Postoperative			
Segmental curve(°)	32.5 ± 11.2 (17-58)	26.0 ± 5.2 (18–46)	0.076
Main curve(°)	36.5 ± 10.4 (20-58)	30.2 ± 5.1 (25–41)	0.405
IM corr (%)	69.3 ± 12.2(51.3-84.2)	77.3 ± 10.5(55.6-87.5)	0.727
Compensatory cranial curve(°)	27.5 ± 5.4 (18-36)	17.8 ± 4.3 (15–28)	0.030
IM corr (%)	68.4 ± 13.3(55.4-90.5)	70.3 ± 14.2(62.1–91.3)	0.546
Compensatory caudal curve (°)	21.9 ± 4.9 (15-32)	17.4 ± 4.1 (12-25)	0.504
IM corr (%)	63.1 ± 11.4(57.3-88.2)	71.2 ± 12.1(55.6–92.1)	0.421
Coronal balance(cm)	0.3 ± 1.0(−1.2 to 1.6)	0.4 ± 0.8(−1.3 to 1.5)	0.590
Sagittal balance(cm)	0.2 ± 1.1(−1.0 to 3.0)	0.7 ± 1.2(−2.3 to 2.8)	0.158
T5-12 kyphosis(°)	32.7 ± 9.7(22-64)	35.5 ± 9.8(27–58)	0.359
L1-S1 lordosis(°)	−43.6 ± 7.2(36-59)	−46.5 ± 6.6(38–56)	0.197
Final follow-up			
Segmental curve(°)	33.2 ± 10.8(18-55)	27.2 ± 6.4(12–50)	0.065
Main curve(°)	38.3 ± 11.2 (16-60)	31.2 ± 6.1 (20–48)	0.115
Final corr (%)	65.4 ± 13.3(48.6-86.2)	74.2 ± 11.7(52.3-84.2)	0.724
Compensatory cranial curve(°)	30.5 ± 6.2 (20-41)	18.2 ± 5.5 (18–32)	0.017
Final corr (%)	64.7 ± 12.5(52.3-91.7)	66.4 ± 10.9(55.8–93.8)	0.437
Compensatory caudal curve(°)	23.2 ± 4.1 (14-30)	18.5 ± 6.2 (14–28)	0.426
Final corr (%)	58.3 ± 11.7(48.3-77.4)	70.2 ± 12.5(53.4–90.2)	0.512
Coronal balance(cm)	0.4 ± 1.2(−1.4 to 1.2)	0.2 ± 0.6(−1.3 to 1.7)	0.768
Sagittal balance(cm)	0.1 ± 1.4(−1.5 to 3.3)	0.6 ± 1.4(−2.2 to 2.4)	0.250
T5-12 kyphosis(°)	34.2 ± 8.6(20-66)	33.2 ± 7.8(26–60)	0.334
L1-S1 lordosis(°)	44.2 ± 5.8(38-61)	43.5 ± 6.3(35–59)	0.243

Significant differences were found in compensatory cranial curve correction between the 2 groups. The postoperative mean angle of compensatory cranial curve was 27.5 ± 5.4 in group A and 17.8 ± 4.3 in group B (*P* = 0.030). At the final follow-up, the mean angle of compensatory cranial curve in group B remained less than in group A (30.5 ± 6.2 in group A vs.18.2 ± 5.5 in group B, *P* = 0.017).However, though there are significant differences in postoperative compensatory cranial curve.

### Complications

Perioperative complications included pedicle failure (1 patient, group A), soft-tissue pain (1 patient, group B), and implant failure (1 patient, group B). There were no neurological complications. One patient in group B experienced implant failure approximately 3 weeks after the internal distraction surgery. The patient did not have any neurological compromises. One patient complained of soft-tissue pain in group A on the concave side of the spine after internal distraction surgery. The pain was relieved in approximately 7 d post-surgery through infrared therapy.

## Discussion

Correction of multi-level severe congenital scoliosis is challenging because of the high rate of associated neurological complications. Procedures used to treat severe rigid congenital scoliosis are fusion in situ, convex-side growth arrest (epiphysiodesis), and hemivertebra resection [12–16]; however, angular osteotomies, including Ponte, Smith–Peterson, and pedicle subtraction, might not provide the desired amount of correction in very severe deformities with coronal or sagittal plane decompensation.

Over the last 10 years, PVCR has been described and provides a positive correction effect [2, 3, 7, 17]. In spite of the positive results in some severe scoliosis, a high risk of more blood loss, neurological damage and instrumentation failure and high technique demanding partly limits wide use of PVCR. Chang et al. [18] reported on 45 patients with congenital scoliosis who were younger than 18 years undergoing posterior vertebral resection and had a complication rate of 48.9%. Wang et al. [17] reported that there are 30.7% patients suffering neurological complications in multilevel PVCR for treating spinal deformity. Shimode et al. [19] reported transient leg paresis in 29% (2 of 7) in PVCR surgery in treating spinal deformity.

Some authors used the growing rod technique in treating congenital scoliosis and gained positive corrective results. Wang et al. [10] performed one-stage posterior osteotomy with short segmental fusion and the dual growing rod technique in 7 patients with severe rigid congenital scoliosis and experienced positive corrective results. Buchowski et al. [20] reported an average correction of 80% by posterior or anterior release and internal distraction followed by posterior spinal fusion with definitive dual-rod fixation, although curvatures <90°were included in that series. Tan et al. [21] used less-invasive internal distraction as an aid to correct severe scoliosis in 15 patients. The average preoperative major curve magnitude was 129.4°. The total major coronal curve correction was 81.4° or 62.9%.

As long-segment congenital scoliosis is often associated with low weight of patients, it’s important to find the most effective method. Anterior release, and posterior release with PSO and posterior vertebral column resection (PVCR), are both feasible means of treating severe scoliosis. However, anterior release may damage the stability of local segments in patients with severe congenital scoliosis. To the best of our knowledge, ours is the first study to have compared posterior release, internal distraction, final PSO and spinal fusion with one-stage PVCR in the treatment of multi-level severe congenital scoliosis. The differences between the two groups in restoring sagittal andcoronal balance were not significant statistically (Table 3). Group B patients had greater postoperative and final curve correction and compensatory cranial and caudal curve than patients in group A. Longer hospital stays, and sometimes more surgeries for patients in group B contributed the most to the differences in hospital expenses between the 2 groups. Our results show that posterior release and growing rod expansion technique has the similar effect with PVCR on correcting main bending and lower compensatory bending, but has a better effect on correcting the upper compensatory bending.

The distraction operation takes advantage of the viscoelastic properties of the spine and allows maximum correction of the deformity with minimal stress on the tissues and implants, which may decrease the rates of implantation failure.

Particularity of long-segment congenital scoliosis is that vertebral structures grow abnormally. If anterior release is conducted but no fixation is taken, local instability may occur to the operation when the patient is required to be turned around for the posterior surgery, damaging the spinal nerve. Thus, the posterior release and growing rod expansion technique is applied to reduce the influence of anterior release operation on pulmonary function of patients with long-segment scoliosis. To the best of our knowledge, this isthe first comparative report between posterior release, internal distraction, final PSO and spinal fusion with one-stage posterior vertebral column resection in treating multi-level severe congenital scoliosis. Previous studies showed that the posterior musculature plays an important role in the formation and development of scoliosis [22, 23]. The use of posterior release with the growing rod may give the spinal cord time to adapt, avoiding nerve damage caused by nerve traction in correction surgery.

Our study found that posterior release, internal distraction, final PSO and spinal fusion can achievethe similar major curve correction rate as well as sagittal and coronal plane balance recovery with PVCR. However, considering smaller complications of nerve injury, less blood loss and easier operation, PSO appears to be a better choice for the correction of multi-level severe congenital scoliosis.

This paper has some disadvantages. First, this is a retrospective study, so the evidence is therefore not as compelling as the prospective studies. Second, the number of patients was small. Further study with a large number of cases will be needed to confirm our results. Third, choosing of surgical methods just based on each surgeon’s judgment can cause selection bias. Unfortunately, we have not found out any definite factors influencing surgeon’s judgment, such as the patent’s age, body weight or main curve of congenital scoliosis. In addition, our patients had cervicothoracic compensatory scoliosis, but no significant cervicothoracic kyphosis. All patients had a good range of neck flexion and extension, so we did not measure chin brow vertical angle or other parameters. Further studies are needed to establish the best means of treating patients with severe cervicothoracic kyphosis.

As a whole, this paper firstly compares results of two operations. We believe that the group of patients who receive posterior release and growing rod technique have their upper compensatory bending significantly improved. Compared with PVCR surgery, this technique can reduce risk of neurological injury and complicating diseases and may be an ideal choice for patients with severemulti-level congenital scoliosis.

## Conclusions

We comparedradiologic results of posterior release, internal distraction, and final spinal fusion with one-stage posterior vertebral column resection (PVCR) in treating multi-level severe congenital scoliosis. Posterior release, internal distraction, and final spinal fusion produce better corrective results in compensatory cranial curve correction than PVCR in treating severe multi-level congenital scoliosis. We thusrecommend this procedure for the treatment ofmulti-level severe congenital scoliosis.

## Abbreviations

CT: Computed tomography
ICC: The intra-class correlation coefficient
MRI: Magnetic resonance imaging
PSO: Pedicle subtraction osteotomy
PVCR: Posterior vertebral column resection
